# Fat and lean mass association with osteoporosis and bone mineral density: the ELSA-Brasil cohort

**DOI:** 10.1210/jendso/bvag119

**Published:** 2026-05-20

**Authors:** Thiago Bosco Mendes, Almog Shalit, Diogo Souza Domiciano, Giuliano Generoso, Virgínia Lúcia Nazário Bonoldi, Valéria de Falco Caparbo, Isabela M Bensenor, Paulo Andrade Lotufo, Marcio Sommer Bittencourt, Pouneh K Fazeli

**Affiliations:** Division of Endocrinology, University of Pittsburgh Medical Center, Pittsburgh, PA 15213, USA; Division of Endocrinology, University of Pittsburgh Medical Center, Pittsburgh, PA 15213, USA; Bone Metabolism Laboratory, Division of Rheumatology, University of São Paulo, São Paulo, SP 01246-903, Brazil; Center for Clinical and Epidemiological Research, University of São Paulo, São Paulo, SP 05508-000, Brazil; Bone Metabolism Laboratory, Division of Rheumatology, University of São Paulo, São Paulo, SP 01246-903, Brazil; Bone Metabolism Laboratory, Division of Rheumatology, University of São Paulo, São Paulo, SP 01246-903, Brazil; Center for Clinical and Epidemiological Research, University of São Paulo, São Paulo, SP 05508-000, Brazil; Center for Clinical and Epidemiological Research, University of São Paulo, São Paulo, SP 05508-000, Brazil; Center for Clinical and Epidemiological Research, University of São Paulo, São Paulo, SP 05508-000, Brazil; Division of Cardiology, University of Pittsburgh Medical Center, Pittsburgh, PA 15213, USA; Division of Endocrinology, University of Pittsburgh Medical Center, Pittsburgh, PA 15213, USA; Center for Human Integrative Physiology, University of Pittsburgh School of Medicine, Pittsburgh, PA 15213, USA

**Keywords:** osteoporosis, bone density, lean mass, fat mass, body composition, sex differences

## Abstract

**Context:**

The relationship between body composition, especially fat mass (FM), and bone mineral density (BMD) is still a matter of debate.

**Objective:**

The aim of this study was to test whether FM and lean mass (LM) are predictors of osteoporosis and if the association between body composition and BMD varies by sex, age, and body mass index (BMI) status.

**Methods:**

We analyzed 1316 participants from the ELSA-Brasil Cohort who underwent dual-energy X-ray absorptiometry. Osteoporosis prevalence was stratified by BMI category and sex. Multivariable regression analyses were performed with lean and FM indexes as predictors of osteoporosis and BMD at different sites according to sex, age, and BMI category.

**Results:**

Osteoporosis was prevalent in 18.6% of postmenopausal females and 8% of males aged ≥50 years. In the fully adjusted model, LM was inversely associated with osteoporosis in the overall male population and in females with BMI ≥30 kg/m^2^ and positively associated with BMD in males and in postmenopausal females with BMI ≥25 kg/m^2^. FM was positively associated with BMD in postmenopausal females with BMI < 25 kg/m^2^.

**Conclusion:**

In postmenopausal women with a BMI < 25 kg/m^2^, BMD is positively associated with FM, whereas in postmenopausal females with a BMI ≥25 kg/m^2^, BMD is positively associated with LM. These findings support the theory that FM is an important predictor of BMD—possibly due to its effect on estradiol levels through aromatization—up until a certain “fat threshold”, after which other factors, including LM and its effect on mechanical loading, likely become more dominant factors affecting BMD.

Osteoporosis, a condition characterized by decreased bone mass and microarchitectural bone tissue deterioration, is typically indolent until progressing to fragility fractures [1]. The disease is prevalent in women older than 65 years, and 50% of postmenopausal females are expected to have a fragility fracture in their lifetime, potentially resulting in significant pain and decreased quality of life [2]. Although less prevalent in males, osteoporosis also has detrimental consequences, and it is projected that 13% of males aged 50 or older will have a fragility fracture in their lifetime [3]. Bone mineral density (BMD) measured by dual-energy X-ray absorptiometry (DXA) is strongly correlated with fracture risk, and it is estimated that for every 1 standard deviation decrease in BMD, fracture risk increases 1.5- to 3-fold [4].

Increased body mass index (BMI) and lean mass are known to be positively associated with BMD [5-10]. However, the impact of fat mass on BMD is still controversial with some studies reporting a neutral or positive association [5-8], and others demonstrating a negative association, especially in men and premenopausal women [10-12]. Mechanically, both lean and fat mass increase bone loading and thereby increase bone mass, with lean mass likely having a greater effect [8]. Adipose tissue effects on bone metabolism, however, are still uncertain [13]. It is known that fat positively affects bone through the expression of aromatase, an enzyme that converts androgens into estrogens, and possibly through increased leptin [13, 14]. It is also known that adipose tissue may negatively affect bone metabolism through the production of proinflammatory molecules, such as interleukin-6 [13].

Epidemiological studies assessing the relationship between fat and lean mass with BMD have considerable variation. This heterogeneity comes from different BMD sites assessed, use of different measures of fat and lean mass (percentage, raw numbers, and/or indexes), adjustment for confounders, statistical methods (correlation versus regression, inclusion of colinear variables in the same model), and population, particularly regarding sex, age, and BMI [5-8, 10-12, 15, 16]. Notably, the relationship between fat mass and BMD differs according to BMI and age in a Korean population, with a greater positive effect on BMD observed in older individuals with a normal BMI [7]. Importantly, lean mass and fat mass are often assessed as a raw number, which fails to adequately account for differences in body size and stature. Conversely, normalizing mass by height squared (kg/m^2^) allows for standardization and consistent comparisons [17].

As the relationship between lean and fat mass and BMD/osteoporosis is still unclear, we looked at this association in individuals from the ELSA-Brasil cohort who underwent DXA. We assessed whether fat and lean mass indices are predictors of osteoporosis. Additionally, we tested the hypothesis that the association between fat and lean mass and BMD varies based on sex, age, and BMI.

## Methods

### Study design and population

The design of the Brazilian Longitudinal Study of Adult Health (ELSA-Brasil) has been previously published [18, 19]. Briefly, 15 105 active or retired University employees aged 35-74 years were voluntarily recruited from six Brazilian cities. Enrollment occurred between 2008 and 2010, and individuals have been followed annually by telephone and with in-person visits from 2012 to 2014 (second visit) and 2017 to 2019 (third visit). In 2017, DXA assessments were initiated at the São Paulo site. Of the 5061 individuals enrolled at this site, 1316 completed DXA measurements between 2017 and 2019 and are included in this study. Data presented in this study refers to the 3rd visit.

### Data collection

Demographic and social characteristics were collected during interviews and clinical examinations. According to the Brazilian Census, race was self-identified as White, Brown (Mixed), Black, Asian, and Indigenous. Smoking was based on self-report in the 3rd visit and classified as never, former, or current use. Physical activity was also self-reported in the 3rd visit and classified as inactive, insufficient (< 150 minutes/week) or active (≥ 150 minutes/week); intense physical activity minutes were multiplied by 2. Alcohol use was self-reported as never, former, or current use. Females self-reported age at menarche, number of pregnancies, and use of hormonal contraceptives at any point. Menopause status was assigned to females who reported “yes” for natural menopause, or “yes” for current or previous use of hormone replacement therapy, or who were 60 years of age or older.

### Dual-energy X-ray absorptiometry

BMD at the hip, femoral neck, and spine (L1-L4), and whole body was obtained by DXA using a GE Lunar iDXA with a maximum weight of 204 kg, performed by the same experienced technologist. Body composition was also obtained and divided into 3 compartments: fat mass, lean mass, and bone mineral content.

Following the International Society for Clinical Densitometry (ISCD) criteria, osteoporosis was defined as a T score less than or equal to –2.5 at the lumbar spine, femoral neck, or total hip in postmenopausal females and in males aged 50 or older, based on the NHANES III population, as there is no Brazilian reference database [20, 21]. The mean interval between DXA and the 3rd visit was 8.3 months [interquartile range: .26, 17].

### Anthropometric measures

Body weight was calculated as the sum of DXA measurements of lean mass, fat mass, and bone mineral content. Height was obtained by a wall stadiometer (Seca, Hamburg, BRD) with a precision of 1 mm, while volunteers were touching their head, buttocks, and heels to the wall and looking straight ahead in the horizontal plane. Lean mass index (LMI) was calculated as lean mass in kg divided by height squared (m^2^), fat mass index (FMI) as fat mass in kg divided by height squared (m^2^), and BMI as weight in kg divided by height squared (m^2^).

### Statistical analyses

All statistical analyses were performed using Stata (StataCorp. 2025. Stata 19 ®, College Station, TX) for Mac. Normality distribution was checked, and baseline variables with normal distribution are presented as mean ± standard deviation (SD); the non-normal variable is reported as median with interquartile range [IQR 25, 75th].

Osteoporosis prevalence was calculated with respect to sex and BMI. Logistic regression analyses with LMI and FMI as predictors of osteoporosis were then performed, accounting for the following confounders: age, race, tobacco use, alcohol use, and physical activity. In females, three additional potential confounders were included in the models: age at menarche, number of pregnancies, and history of hormonal contraceptive use. Stratification for BMI categories was then performed.

Linear regression analyses were initially performed to check for LMI and FMI as predictors of BMD at different sites. A regression including both LMI and FMI in the same model was then carried out, followed by a multivariate analysis including LMI, FMI, age, and sex. The fully adjusted linear regression model included age, sex, race, tobacco use, physical activity, and alcohol use, with the addition of number of pregnancies, age at menarche, and use of hormonal contraceptives for females. The marginal effects of LMI and FMI, divided by quartiles, on BMD were plotted for males and females. Further linear regression analyses with stratification by sex, BMI category, and age ≥ 50 years for males and menopause for females were then executed. For females, the BMI cutoff was 25 kg/m^2^. As there were only 11 males younger than 50 years old with a BMI < 25 kg/m^2^, a BMI cutoff of 30 kg/m^2^ was used for them. Variance inflation factor (VIF) was calculated to ensure that collinearity was not present, and BMI was not placed in the above models for this reason.

Results are presented as frequencies and percentages for categorical variables and coefficients for the linear regression analyses. Individuals were excluded from the model if one or more of the variables were missing. In the logistic regression, individuals were also excluded if a level of the variable perfectly predicted failure in the model. A two-sided *P* level ≤ .05 was considered statistically significant.

### Ethical considerations

The study was approved by the Brazilian National Research Ethics Committee (CONEP140/08) and by the ethics committees of all participating institutions. All participants provided informed consent before enrollment.

## Results

### Baseline characteristics

A total of 1316 individuals with a mean age of 58 ± 8.5 years were assessed, of which 61.2% were female. Most Individuals self-reported themselves as White, Brown (Mixed), or Black: 59.2%, 22%, and 12.6%, respectively. The mean BMI was 28.3 kg/m^2^ and 51.4% of the women were postmenopausal (Table 1).

**Table 1:** Baseline characteristics

	Sex		
	Female	Male	Total
Number of individuals	805 (61.2%)	511 (38.8%)	1316(100.0%)
Age (years)	58.1 ± 8.1	57.9 ± 9.1	58 ± 8.5
Race			
White	471 (58.8%)	301 (59.7%)	772 (59.2%)
Brown (mixed)	165 (20.6%)	122 (24.2%)	287 (22.0%)
Black	108 (13.5%)	57 (11.3%)	165 (12.6%)
Asian	52 (6.5%)	19 (3.8%)	71 (5.4%)
Indigenous	5 (.6%)	5 (1.0%)	10 (.8%)
Tobacco use			
Former	232 (28.9%)	185 (36.3%)	417 (31.8%)
Currently	83 (10.3%)	47 (9.2%)	130 (9.9%)
Alcohol use			
Former	109 (15.7%)	63 (13.5%)	172 (14.8%)
Currently	445 (63.9%)	378 (80.9%)	823 (70.8%)
Physical activity			
Inactive	146 (18.4%)	88 (17.5%)	234 (18.1%)
Insufficient	258 (32.6%)	135 (26.9%)	393 (30.4%)
Active	388 (49.0%)	279 (55.6%)	667 (51.5%)
Weight (kg)	71.3 ± 15.2	82.8 ± 14.3	75.7 ± 15.8
BMI (kg/m^2^)	28.4 ± 5.5	28.2 ± 4.3	28.3 ± 5.1
Lean mass (kg)	39.4 ± 5.9	54 ± 7.4	45.1 ± 9.7
Lean mass index (kg/m^2^)	15.7 ± 2	18.4 ± 2	16.8 ± 2.4
Fat mass (kg)	29.7 ± 10.1	25.9 ± 8.3	28.2 ± 9.6
Fat mass index (kg/m^2^)	11.8 ± 3.9	8.84 ± 2.8	10.7 ± 3.8
Menarche	12.7 ± 1.7	—	12.7 ± 1.7
Number of pregnancies	2 [1,3]	—	2 [1,3]
Hormonal contraceptive (current/past)	613 (76.1%)	—	613 (76.1%)
Postmenopausal	414 (51.4%)	—	414 (51.4%)

Data presented as number (percentage), mean ± standard deviation, and median [interquartile range].

### Osteoporosis prevalence by sex and BMI

**Figure 1:**
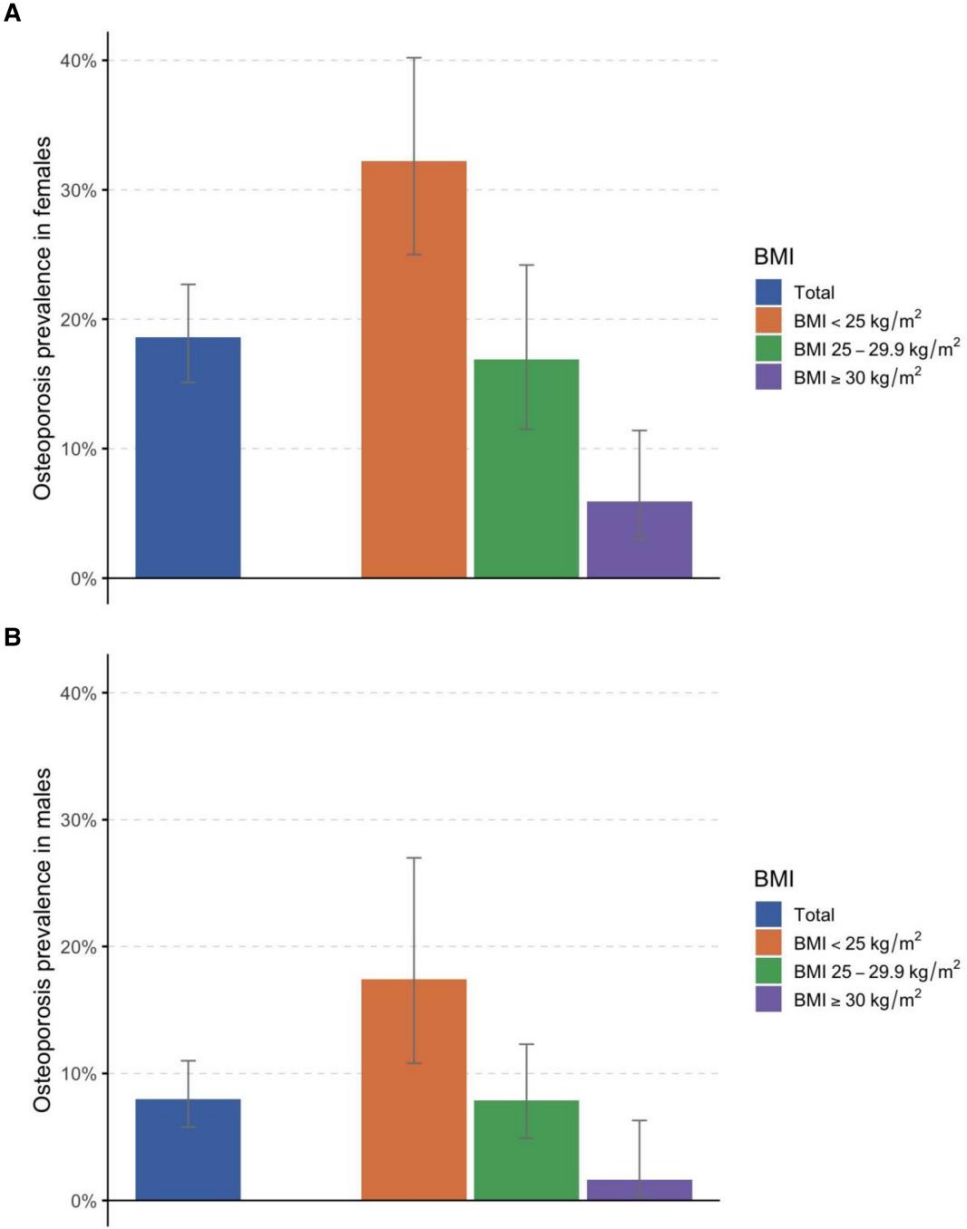
Osteoporosis prevalence in (A) females and (B) males, stratified by obesity status.

Seventy-seven of the 414 postmenopausal females (18.6%; 95% confidence interval [CI]: 15.1-22.7%) met criteria for osteoporosis based on DXA results. In postmenopausal females with BMI < 25 kg/m^2^, osteoporosis prevalence was 32.2% (95% CI: 25-40.2%), while it was 16.9% (95% CI: 11.5-24.2%) for those with BMI between 25 and 29.9 kg/m^2^, and 5.93% (95% CI: 2.98-11.4%) for BMI ≥ 30 kg/m^2^ (Fig. 1A).

Of the 424 males aged 50 years and older, osteoporosis was present in 34 (8%; 95% CI: 5.78-11%). For those with BMI < 25 kg/m^2^, the prevalence was 17.4% (95% CI: 10.8-27.1%) while it was 7.9% (95% CI: 4.9-12.3%) in those with BMI between 25 and 29.9 kg/m^2^. Out of 100 males aged 50 and older with BMI ≥ 30 kg/m^2^, only 2 had osteoporosis (1.64%, 95% CI: 0.4-6.3%), both with a BMI of 30.1 kg/m^2^ (Fig. 1B).

### LMI and FMI as predictors of osteoporosis by sex and BMI status

In the fully adjusted model, FMI was associated with lower odds of osteoporosis in postmenopausal females, with an OR of .85 (95% CI: 0.74-0.98, *P*: .02) per 1 incremental kg/m^2^, whereas the OR for LMI was .79 (95% CI: 0.61-1.03, *P*: .09). For males aged 50 years and older, LMI was associated with lower odds of osteoporosis with an OR of .68 (95% CI: 0.5-0.92, *P*: .01) per 1 incremental kg/m^2^, whereas the OR for FMI was .9 (95% CI: 0.72-1.25, *P*: .36) (Table 2).

**Table 2:** Adjusted logistic regression of LMI and FMI as predictors of osteoporosis in females and males stratified by BMI

	N	Odds ratio (95% confidence interval)
**Females—overall population**		
LMI	342	.79 (.61, 1.03)
**FMI**		**.85** (.74, .98)*
**Females—BMI < 25 kg/m^2^**		
LMI	115	.78 (.47, 1.28)
FMI		**.69** (.48, .99)*
**Females—BMI between 25 and 29.9 kg/m^2^**		
LMI	92	1.66 (.8, 3.44)
FMI		.8 (.47, 1.36)
**Females—BMI ≥ 30 kg/m^2^**		
LMI	108	**.31** (.1, .95)*
FMI		.96 (.58, 1.59)
**Males—overall population**		
LMI	379	**.68** (.5, .92)*
FMI		.9 (.72, 1.12)
**Males—BMI < 25 kg/m^2^**		
LMI	51	.78 (.45, 1.34)
FMI		1.18 (.72, 1.96)
**Males—BMI between 25 and 29.9 kg/m^2^**		
LMI	192	.75 (.41, 1.36)
FMI		.87 (.53, 1.43)
**Males—BMI ≥ 30 kg/m^2^**		
LMI	Not calculated as there were only 2 individuals with osteoporosis	
FMI		

BMI, body mass index; FMI, fat mass index; LMI, lean mass index.

Adjusted for age, race, tobacco, alcohol use, physical activity, and FMI in the model for LMI and LMI in the model for FMI. For females: the model was also adjusted for age of menarche, number of pregnancies and hormone replacement therapy. Observations with missing covariate(s) or covariate(s) that perfectly predicted failure were excluded from the model.

*P < .05.

Upon stratification by BMI, for postmenopausal women with BMI < 25 kg/m^2^, FMI was independently associated with lower odds of osteoporosis with an OR of .69 (95% CI: 0.48-0.99, *P*: .04), whereas for those with BMI ≥ 30 kg/m^2^, LMI was independently associated with lower odds of osteoporosis with an OR of .31 (95% CI: 0.1-0.95, *P*: .04) in the fully adjusted model (Table 2).

For males aged 50 and older, LMI was associated with lower odds of osteoporosis, and stratification by BMI was not possible as only two males with BMI ≥ 30 kg/m^2^ had osteoporosis (Table 2).

### LMI and FMI as positive predictors of BMD in the overall population

**Figure 2:**
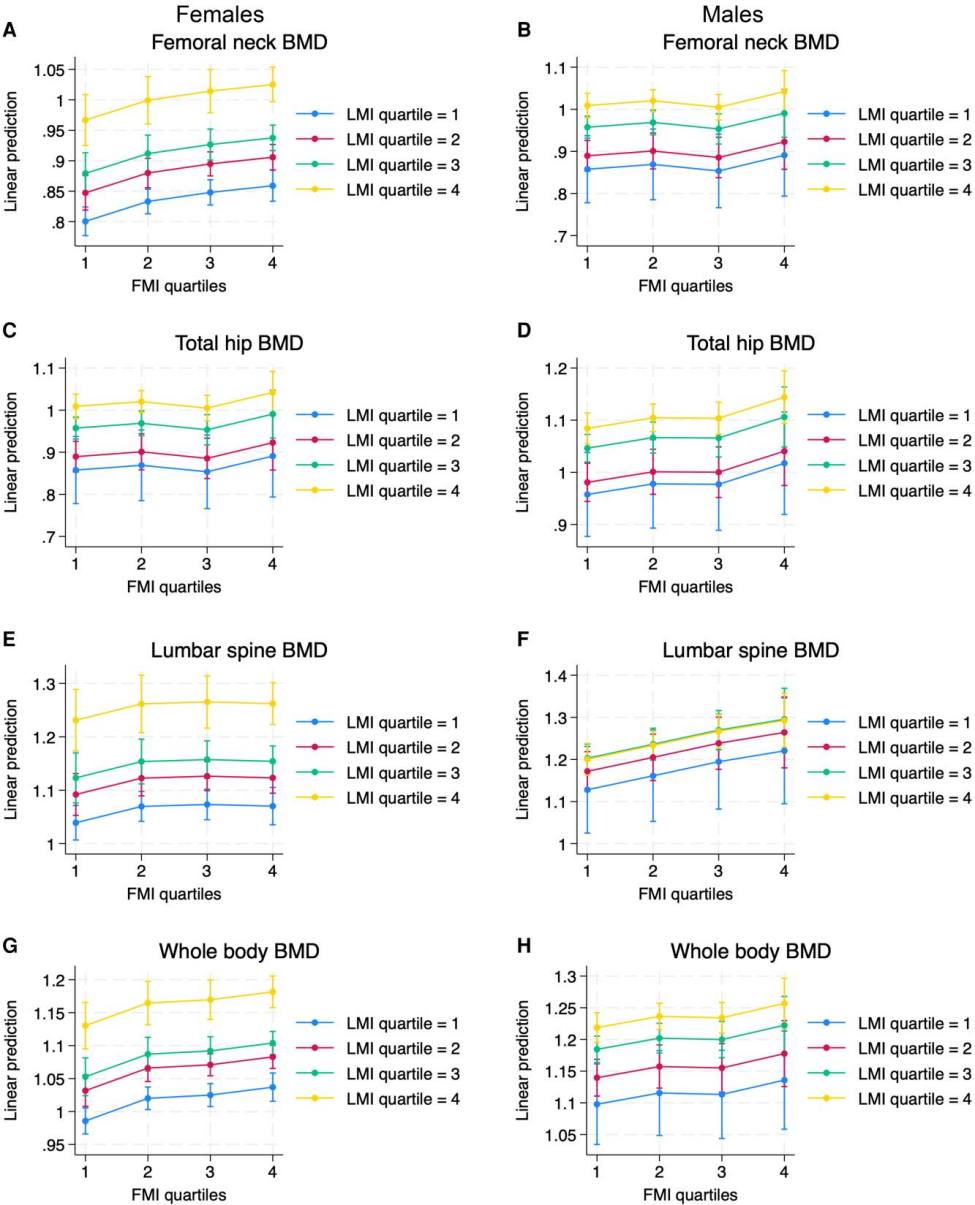
Adjusted marginal effect of quartiles of LMI and FMI on BMD (g/cm^2^) stratified by sex. A, femoral neck in females; B, femoral neck in males; C, total hip in females; D, total hip in males; E, lumbar spine in females; F, lumbar spine in males; G, whole body in females; H, whole body in males. Adjusted for age, race, tobacco, alcohol use, and physical activity. For females, the models are also adjusted for age at menarche, number of pregnancies, and current or previous use of hormonal contraceptive.

In unadjusted linear regression analyses, both LMI and FMI were positively associated with BMD (g/cm^2^) at all sites, with LMI having higher coefficients (Table 3). When LMI and FMI were included in the same model (Model 1), LMI continued to be positively associated with BMD, while FMI was either no longer associated or was associated with lower BMD (Table 3). Further adjustments for age and sex (Model 2) revealed that both LMI and FMI were positively associated with BMD, with LMI presenting with higher coefficients (Table 3). In the fully adjusted model (Model 3), both LMI and FMI were positively associated with BMD at different sites, with LMI having higher coefficients (Table 3). The marginal effects of LMI and FMI on BMD indicate how LMI and FMI prediction of BMD (g/cm^2^) is variable according to BMD site and sex (Fig. 2).

**Table 3:** Linear regression coefficients (95% confidence interval) for BMI, LMI, and FMI as predictors of BMD (g/cm^2^) for the overall population

	N	Femoral neck BMD	Total hip BMD	L1-L4 BMD	Whole body BMD
**Unadjusted**					
BMI	1316	.0104 (.0090, .0119)*	.0125 (.0110, .0140)*	.0100 (.0081, .0120)*	.0092 (.0078, .0106)*
LMI	1316	.0304 (.0275, .0334)*	.035 (.0320, .038)*	.0294 (.0254, .0334)*	.0333 (.0307, .0358)*
FMI	1316	.0058 (.0037, .0079)*	.0076 (.0054, .0098)*	.0051(.0024, .0078)*	.0022 (.0002, .0042)***
**Model 1**					
BMI*^a^*	Not applicable				
LMI	1316	.0303 (.0273, .0334)*	.0344 (.0313, .0375)*	.0296 (.0254,.0337)*	.0353 (.0326, .038)*
FMI	1316	.0002 (−.0017, .0021)	.0012 (−.0007, .0032)	−.0003 (−.003, .0023)	−.0043 (−.006, −.0027)*
**Model 2**					
BMI*^a^*	1316	.0099 (.0085, .0112)*	.0121 (.0107, .0135)*	.0099 (.008, .0117)*	.0088 (.0076, .0099)*
LMI	1316	.0234 (.0188, .0279)*	.0261 (.0214, .0308)*	.016 (.0097, .0225)*	.0178 (.014, .02165)*
FMI	1316	.0029 (.0003, .0054)***	.0048 (.0022, .0074)*	.0062 (.0026, .0098)**	.0038 (.0017, .006)**
**Model 3**					
BMI*^a^*	1140	.0096 (.0081, .0111)*	.0119 (.0103, .0134)*	.0097 (.0077, .0119)*	.0084 (.0071, .0097)*
LMI	1140	.0214 (.0163, .0265)*	.024 (.0188, .0292)*	.0154 (.0082,.0225)*	.0168 (.0126, .0211)*
FMI	1140	.0034 (.0006, .0063)***	.0055 (.0026, .0085)*	.0064 (.0024,.0104)**	.0038 (.0014, .0062)**

BMD, bone mineral density; BMI, body mass index; FMI, fat mass index; LMI, lean mass index; N, number of individuals.

Model 1: FMI adjusted for LMI and LMI adjusted for FMI. BMI not in the model due to collinearity.

Model 2: adjusted for age and sex. In the FMI model also adjusted for LMI; in the LMI model also adjusted for FMI.

Model 3: adjusted for age, sex, race, tobacco, alcohol use, physical activity, and FMI in the model for LMI and LMI in the model for FMI. Observations with missing covariate(s) were excluded from the model.

^*a*^Not adjusted for LMI or FMI due to collinearity.

*P < .001.

**P < .01.

***P < .05.

### LMI and FMI as predictors of BMD stratified by age, sex, and BMI

In postmenopausal females with BMI < 25 kg/m^2^, LMI was only positively associated with whole body BMD (g/cm^2^), whereas FMI was positively and independently associated with BMD at the femoral neck, total hip, and whole body in the fully adjusted model. For postmenopausal females with BMI ≥ 25 kg/m^2^, LMI was associated with higher BMD at all sites, whereas FMI was not associated with BMD. Data were available for 86 premenopausal females with BMI < 25 kg/m^2^, and there was no association between LMI or FMI with BMD in this group. For premenopausal females with BMI ≥ 25 kg/m^2^, LMI was positively associated with BMD at all sites, whereas FMI was not associated with BMD (Table 4).

**Table 4:** Adjusted linear regression coefficients (95% confidence interval) for LMI and FMI as predictors of BMD (g/cm^2^) stratified by sex, BMI and menopausal status for females, and age ≥ 50 years or < 50 years for males

	N	Femoral neck BMD	Total hip BMD	L1-L4 BMD	Whole Body BMD
**Postmenopausal females with BMI < 25 kg/m^2^**					
LMI	116	.0121 (−.0096, .0338)	.0175 (−.0063, .0413)	.0362 (−.0009, .0733)	.0217 (.0002, .0431)***
FMI	116	.0176 (.0026, .0326)***	.0305 (.0141, .047)*	.0165 (−.0091, .0421)	.02 (.0052, .0348)**
**Postmenopausal females with BMI ≥ 25 kg/m^2^**					
LMI	225	.0183 (.0071, .0294)**	.0239 (.0119, .0359)*	.0225 (.007, .038)**	.0123 (.0027, .022)***
FMI	225	.0006 (−.005, .0061)	.0012 (−.0048, .0072)	.004 (−.0038, .0117)	.0034 (−.0015, .0082)
**Premenopausal females with BMI < 25 kg/m^2^**					
LMI	86	.0135 (−.0157, .0427)	.0165 (−.0132, .0462)	.0179 (−.0172, .0529)	.0129 (−.0095, .0353)
FMI	86	−.0015 (−.0192,.0163)	.0012 (−.0168, .0193)	−.0065 (−.0278, .0148)	−.0083 (−.0219, .0053)
**Premenopausal females with BMI ≥ 25 kg/m^2^**					
LMI	252	.022 (.0117, .0323)*	.0314 (.0213, .0415)*	.028 (.0144, .0416)*	.0255 (.0172, .0337)*
FMI	252	.0042 (−.0012, .0096)	.0049 (−.0004, .0102)	−.0016 (−.0088, .0055)	.0006 (−.0037, .0049)
**Males aged 50 years or older with BMI < 30 kg/m^2^**					
LMI	271	.0232 (.0104, .036)*	.0199 (.0067, .0331)**	.0007 (−.0173, .0187)	.0134 (.0026, .0242)***
FMI	271	−.0013 (−.0115, .0089)	.0071 (−.0035, .0176)	.0155 (.0011, .0299)***	.0064 (−.0023, .015)
**Males aged 50 years or older with BMI ≥ 30 kg/m^2^**					
LMI	109	.0268 (.0067, .0469)***	.0279 (.0084, .0475)**	−.0004 (−.026, .0251)	.0128 (−.0021, .0276)
FMI	109	−.0068 (−.0207, .0071)	−.005 (−.0185, .0086)	−.0015 (−.0192, .0162)	−.0026 (−.0129, .0077)
**Males younger than 50 years old with BMI < 30 kg/m^2^**					
LMI	51	.0191 (−.017, .0552)	.0244 (−.0135, .0623)	.0297 (−.0086, .068)	.0296 (.0039, .0552)***
FMI	51	.0169 (−.0057, .0396)	.0161 (−.0077, .0398)	.0229 (−.0011, .0469)***	.0121 (−.004, .0282)
**Males younger than 50 years old with BMI ≥ 30 kg/m^2^**					
LMI	23	.0512 (.0043, .0982)***	.0512 (.0117, .0908)***	.0153 (−.0356, .0662)	.0432 (.0081, .0784)***
FMI	23	−.0135 (−.0412, .0142)	−.0096 (−.0329, .0138)	.001 (−.0291, .0311)	−.0098 (−.0306, .011)

Adjusted for age, race, tobacco, alcohol use, physical activity, and FMI in the model for LMI and LMI in the model for FMI. For females: + age of menarche, number of pregnancies and current or previous use of hormonal contraceptive. Observations with missing covariate(s) were excluded from the model.

*P < .001.

**P < .01.

***P < .05.

For males aged 50 years and older with BMI < 30 kg/m^2^, LMI was independently and positively associated with BMD at 3 out of the 4 sites, whereas FMI was positively associated with BMD only at the lumbar spine. For males aged 50 years and older with BMI ≥ 30 kg/m^2^, LMI independently predicted BMD at the femoral neck and total hip. In males younger than 50 years old and BMI <30 kg/m^2^, data were available for 51 individuals, and LMI was positively associated with whole body BMD, whereas FMI was positively associated with BMD at the lumbar spine. Only 23 males younger than 50 years old with BMI ≥ 30 kg/m^2^ were assessed. In this subgroup, LMI was positively associated with BMD, while there was no association between FMI and BMD.

## Discussion

In the large and diverse ELSA-Brasil cohort, osteoporosis prevalence was as high as 32.2% in postmenopausal females with BMI < 25 kg/m^2^ and as low as 1.64% in males ≥ 50 years old with BMI ≥ 30 kg/m^2^. Fat mass was independently associated with lower odds of osteoporosis in postmenopausal females; an effect attributed to its positive association with BMD in postmenopausal females with BMI < 25 kg/m^2^. Lean mass was consistently and independently associated with higher BMD in males and in females with BMI ≥ 25 kg/m^2^, and lower odds of osteoporosis in males as a whole population and females with BMI ≥ 30 kg/m^2^. We have also shown that LMI and FMI association with BMD varies significantly with BMD site.

Despite consistent evidence that BMI and lean mass are associated with higher BMD [5-10], studies investigating the association between fat mass and BMD have less consistent results [5, 8, 10]. Prior studies have shown that fat mass is associated with higher BMD in postmenopausal females [7, 8, 15, 22] and lean mass is associated with higher BMD in premenopausal females [7, 8, 10, 16]. In this study, fat mass was independently and positively associated with BMD and decreased osteoporosis prevalence in postmenopausal females with normal BMI, with no additional beneficial association in females with a higher BMI (≥ 25 kg/m^2^). In contrast, for postmenopausal females with BMI ≥ 30 kg/m^2^, lean mass was not only associated with a higher BMD but also with decreased odds of osteoporosis. This is consistent with a prior Korean study finding that fat mass is positively associated with BMD in women with normal BMI [7] and suggests that fat mass' positive association with BMD may be via aromatization. In postmenopausal women, fat mass becomes a primary source of estrogens rather than the ovaries, as fat tissue converts androgens to estrogens through expression of aromatase enzyme [23] and it is likely that after a certain estrogen level is achieved—a “fat threshold”—additional estrogen becomes less important for BMD and other factors, such as lean mass and its effects on mechanical loading, become more important determinants of BMD.

The hypothesis that the association between fat mass and BMD is mediated through aromatization is further supported by a study evaluating vertebral fracture in postmenopausal females with early breast cancer; the study found that among those receiving aromatase inhibitors, greater fat mass was associated with an increased risk of fragility fractures, whereas among those not receiving aromatase inhibitors, greater fat mass was linked to a reduced fragility fracture risk [24]. A Canadian study also demonstrated that fat mass is positively associated with femoral neck BMD in females almost linearly until a threshold is reached around a BMI of 25 kg/m^2^ and then the association plateaus [25]. Our data suggest that in postmenopausal women with a normal BMI, and therefore a population that has less mechanical loading on their bone, fat mass—and its ability to increase estrogen levels via aromatization [23]—may be a more important factor for BMD than in women with overweight or obesity, in whom sufficient levels of estrogens are achieved and in whom mechanical loading from lean mass is a more important factor. Therefore, our findings are consistent with the theory that fat aromatization modulates the relationship between fat mass and BMD in postmenopausal females. BMD is positively associated with fat mass until a “fat threshold”, after which the fat mass has no additional benefit and lean mass is associated with higher BMD and lower odds of osteoporosis.

It is widely accepted that lean mass is positively associated with BMD in males [8, 10-12, 16]. Despite some studies showing a detrimental effect of fat mass on BMD in males [10, 12], for those with normal BMI it has been shown that fat mass is associated with higher BMD [7]. In our study, lean mass was consistently and independently positively associated with BMD in males, while fat mass had no detrimental effect and was also positively associated with BMD in at least one BMD site for those with BMI < 30 kg/m^2^. In our cohort, osteoporosis was also extremely uncommon in males with BMI ≥ 30 kg/m^2^, an effect most likely due to increased mechanical loading, given the positive association between lean mass and BMD in both younger and older males with a BMI ≥ 30 kg/m^2^. Although estrogen is a critical hormone for bone in males and decreases with aging, its decline is attenuated in men compared to women. Aromatization in males is likely more dependent and limited by aromatase's substrate, testosterone, and less dependent on fat mass [26], in comparison to postmenopausal females. Our findings support the fact that fat mass may be a less important factor with respect to BMD in males.

The association between fat and lean mass with BMD is complex and multifaceted. Discrepant results in the literature in assessing the association of fat and lean mass with BMD are probably related to differences in methodology and stratification. Methodologically, studies differ in BMD sites assessed, adjustment for confounders, and use of different measures of fat and lean mass, including percentages, raw numbers, and/or indexes. In terms of stratification, study populations differ with respect to race, sex, age, and BMI range [5, 7, 8, 10, 12]. In fact, our study shows that the association of lean mass and fat mass with BMD varies significantly depending on how the analyses are done. For instance, a model with lean and fat mass only as predictors of BMD revealed lean mass to be positively associated with BMD, while fat mass had no or a negative association. However, it is known that females have a higher proportion of fat mass [27] and lower BMD [28] compared to males. After adding age and sex to the model, both lean mass and fat mass were positively associated with BMD, with higher coefficients for lean mass. Stratification for BMI and sex leads to even more variable effects, as previously discussed herein.

Strengths of this study include assessment of a large, contemporary, racially diverse, and community-dwelling population; comprehensive adjustment for confounders; use of lean and fat mass indices rather than raw values; stratification by sex, age, and obesity status; and statistical analyses with robust models carefully built to ensure no collinearity. Limitations include the cross-sectional design, small sample size of some subgroups, and assessment of overall lean and fat mass indices, with no stratification by site of lean or fat mass deposition.

## Conclusion

In summary, we show that lean mass is associated with a higher BMD in postmenopausal females with a BMI ≥ 25 kg/m^2^ and males, whereas fat mass is independently associated with a higher BMD in postmenopausal females with a normal BMI. Our study extends the prior literature by providing a better understanding of the granular association between body composition parameters and BMD in a large and mixed population, in addition to offering a potential explanation for the diversity of associations observed between fat mass and BMD.

## Data Availability

Datasets analyzed during the current study are not publicly available but are available from the corresponding author on reasonable request.
